# 2117 Integrating ethics support as culture change in a translational science environment

**DOI:** 10.1017/cts.2018.289

**Published:** 2018-11-21

**Authors:** Bernadette McKinney, Emma Tumilty, Joseph Kotarba

**Affiliations:** 1 University of Texas Medical Branch at Galveston; 2 University of Texas Medical Branch; 3 University of Texas Medical Branch and Texas State University

## Abstract

OBJECTIVES/SPECIFIC AIMS: To outline 4 categories of ethics needs identified at a translational science center. To map how research ethics has been further integrated into the center’s culture in response to these needs. To provide insights into how research ethics can be integrated into the translational team science environment. METHODS/STUDY POPULATION: The Institute for Translational Sciences (ITS) at the University of Texas Medical Branch is studied on an organizational level using polyphonic organizational theory and the results of an ethics needs assessment completed in 2010. RESULTS/ANTICIPATED RESULTS: The results will be a map indicating how research ethics has been further integrated into the culture of the ITS in response to the needs identified to ensure the responsible practice of translational science. DISCUSSION/SIGNIFICANCE OF IMPACT: Successful translational science requires shared understanding of communication and values. Achieving agreement in these areas requires the development of strategies for communicating and reinforcing common goals. Research ethics has often been considered an “add on” rather than a “part of” science. Through integrating ethics into various aspects of translational science, the ITS has taken important steps toward achieving the goal of culture change. The map of how the ITS has integrated ethics into organizational activities and structures will serve as a model for other organizations and institutions.

